# Interactions among variants in *TXA2R*, *P2Y12* and *GPIIIa* are associated with carotid plaque vulnerability in Chinese population

**DOI:** 10.18632/oncotarget.24801

**Published:** 2018-04-03

**Authors:** Xingyang Yi, Jing Lin, Hua Luo, Ju Zhou, Qiang Zhou, Yanfen Wang, Chun Wang

**Affiliations:** ^1^ Department of Neurology, The People’s Hospital of Deyang City, Deyang 618000, Sichuan, China; ^2^ Department of Neurology, The Third Affiliated Hospital of Wenzhou Medical University, Wenzhou 325200, Zhejiang, China; ^3^ Department of Neurology, The Affiliated Hospital of Southwest Medical University, Luzhou 646000, Sichuan, China

**Keywords:** ischemic stroke, carotid atherosclerosis, carotid plaque vulnerability, genetic polymorphism, platelet activation

## Abstract

**Purpose:**

The associations between variants in platelet activation-relevant genes and carotid plaque vulnerability are not fully understood. The aim of the present study was to investigate the associations of the variants in platelet activation-relevant genes and interactions among these variants with carotid plaque vulnerability.

**Results:**

There were no significant differences in the frequencies of genotypes of the 11 variants between patients and controls. Among 396 patients, 102 patients had not carotid plaque, 106 had VP, and 188 had SP. The 11 variants were not independently associated with risk of carotid plaque vulnerability after adjusting for potential confounding variables. However, the GMDR analysis showed that there were synergistic effects of gene-gene interactions among *TXA2Rr* s1131882, *GPIIIa* rs2317676 and *P2Y12* rs16863323 on carotid plaque vulnerability. The high-risk interactions among the three variants were associated with high platelet activation, and independently associated with the risk of carotid plaque vulnerability.

**Methods:**

Eleven variants in platelet activation-relevant genes were examined using mass spectrometry methods in 396 ischemic stroke patients and 291controls. Platelet-leukocyte aggregates and platelet aggregation were also measured. Carotid plaques were assessed by B-mode ultrasound. According to the results of ultrasound, the patients were stratified into three groups: non-plaque group, vulnerable plaque (VP) group and stable plaque (SP) group. Furthermore, gene-gene interactions were analyzed using generalized multifactor dimensionality reduction (GMDR) methods.

**Conclusions:**

The rs1131882, rs2317676, and rs16863323 three-loci interactions may confer a higher risk of carotid plaque vulnerability, and might be potential markers for plaque instability.

## INTRODUCTION

Stroke is one of leading causes of mortality and disability in China [1, 2]. Carotid atherosclerosis is a major risk factor for ischemic stroke (IS). Carotid plaque rupture or vulnerable lesions may obstruct the blood vessels of the brain by atherothrombosis or emboli [3, 4].The degree of carotid stenosis alone may not be sufficient to evaluate the risk of stroke [5]. The cholucent plaque or ulcerative plaque in carotid may play a more important role in the occurrence of cerebrovascular events than carotid stenosis [5, 6]. Therefore, identifying novel etiologies of carotid plaque vulnerability, including the genetic etiology is very important for preventing stroke [7]. However, up to date, such a genetic etiology has not been fully understood.

Carotid plaque vulnerability may be assessed by B-mode ultrasound or high-resolution magnetic resonance imaging (HR-MRI), HR-MRI may provide more information regarding plaque composition and morphology [8]. However, HR-MRI is not routinely used to assess carotid plaque vulnerability in China, because of expensive cost of HR-MRI assessment. Clinically, carotid ultrasound is a well method for visualizing and quantifying carotid atherosclerotic lesions and its vulnerability, and echo-lucent lipid-rich plaques are associated with more complications than that of mixed or calcified plaques [9, 10]. Heterogeneous plaques were reportedly associated with intraplaque hemorrhage and ulceration [11].

Atherosclerosis is a complex inflammatory disorder. Platelet activation, inflammation and endothelial functionplay key roles in the atherosclerosis pathogenesis. Gardener *et al.* [12] have reported that the variations of genes involved in endothelial function and inflammation are associated with carotid plaque stability in a Dominican population. Our previous studies showed that genetic variants of Cytochrome P450 and cyclooxygenase pathway genes were associated with plaque stability [13, 14]. Platelet aggregation and platelet-leukocyte aggregates play a key role in thrombogenesis and IS [15, 16], antiplatelet therapy is recommended for prevention of IS [17]. Platelet activation is also associated with the pathophysiology of atherogenesis [18, 19]. Prostaglandin H can be metabolized by thromboxane synthase (TXAS) into thromboxanes A2 (TXA2), a potent vasoconstrictor and platelet activator. TXA2 bind to its receptor TXA2R are associated with higher platelet activation [13]. Platelet membranes receptors (P2Y1, P2Y12) and fibrinogen receptor [glycoprotein IIIa (GPIIIa) and glycoprotein IIb (GPIIb) are the final common pathway of platelet activation, aggregation, and adhesion [20, 21], and play important roles in the process of platelet activation [20]. Previous studies have revealed that single nucleotide polymorphisms (SNPs) in *TXA2R, P2Y1, P2Y12* and *GPIIb/IIIa* genes significantly increase the risk for IS [13, 22], and influence response to antiplatelet drugs [23–25]. However, the associations between variants in platelet activation-relevant genes and plaque vulnerability have not well been underscored. Furthermore, few studies investigated the effect of gene-gene interactions among platelet activation-relevant genes on carotid plaque vulnerability using generalized multifactor dimensionality reduction (GMDR) method [26].

In this study, we hypothesized that the platelet activation-relevant genetic variants and these variants interactions were associated with the risk for carotid plaque vulnerability by influencing platelet activation. To test this hypothesis, we evaluated 11 variants from platelet activation-relevant genes and platelet activation in 396 IS patients and 291 controls to determine the associations of these variants and interactions among these variants with carotid plaque vulnerability in Chinese population.

## RESULTS

### Clinical characteristics, genotype distributions and platelet activation in patients and controls

Diabetes mellitus and hypertension were more frequent in patients than controls (Table1). However, there were no significant differences in other risk factors between the two groups (Table 1). The genotype distributions of the 11 variants were in Hardy–Weinberg equilibrium (*P* > 0.05), and there were no significant differences in genotype distributions of the 11 variants between patients and controls (Table 1). The arachidonic acid (AA) or adenosine diphosphate (ADP)-induced platelet aggregation and platelet-leukocyte aggregates were higher in patients compared with controls (Table 1).

**Table 1:** Clinical characteristics and genotype distributions in patients and controls (*n*, %)

	Stroke patients (*n* = 396)	Controls (*n* = 291)	*P* value
Age (years)	68.4 ± 11.8	66.9 ± 10.9	0.083
Men (*n*, %)	235 (59.3)	165 (56.7)	0.473
Hypertension (*n*, %)	287 (72.5)	130 (44.7)	<0.001
Diabetes mellitus (*n*, %)	138 (34.8)	73 (25.1)	0.006
Body mass index (kg/m^2^)	24.1 ± 2.3	23.9 ± 2.5	0.289
Current smoking (*n*, %)	160 (40.4)	118 (40.5)	0.998
Previous or ongoing drug treatments (*n*, %)			
Antihypertensive drugs	121 (30.6)	75 (25.8)	0.201
Hypoglycemic drugs	95 (23.9)	58 (19.9)	0.225
Statins	51 (12.9)	32 (11.0)	0.398
Antiplatelet drugs	83 (20.9)	47 (16.2)	0.124
*TXA2R* (rs1131882)			
CC	135 (34.1)	100 (34.4)	0.978
CT	184 (46.5)	135 (46.4)	
TT	77 (19.4)	56 (19.2)	
*TXAS1* (rs194149)			
AA	65 (16.4)	40 (13.7)	0.193
AG	197 (49.7)	164 (56.4)	
GG	134 (33.8)	87 (29.9)	
*TXAS1* (rs2267679)			
CC	11 (2.8)	1 (0. 3)	0.106
CT	91 (23.0)	47 (16.2)	
TT	294 (74.2)	243 (83.5)	
*TXAS1* (rs41708)			
GG	239 (60.4)	177 (60.8)	0.764
GT	110 (27.8)	92 (31.6)	
TT	47 (11.9)	22 (7.6)	
*P2Y1* (rs701265)			
AA	217 (54.8)	168 (57.7)	0. 482
AG	119 (30.0)	85 (29.2)	
GG	60 (15.2)	38 (13.1)	
*P2Y1* (rs1439010)			
AA	215 (54.3)	169 (58.1)	0.343
AG	122 (30.8)	84 (28.8)	
GG	59 (14.9)	38 (13.1)	
*P2Y1* (rs1371097)			
CC	208 (52.5)	170 (58.4)	0.265
CT	125 (31.6)	87 (29.9)	
TT	63 (15.9)	34 (11.7)	
*P2Y12* (rs16863323)			
CC	121 (30.6)	73 (25.1)	0.513
CT	131 (33.1)	102 (35.1)	
TT	144 (36.4)	116 (39.8)	
*P2Y12* (rs9859538)			
GG	273 (68.9)	215 (73.9)	0.327
AG	87 (22.0)	58 (19.9)	
AA	36 (9.1)	18 (6.2)	
*GPIIIa* (rs2317676)			
AA	242 (61.1)	186 (63.9)	0.397
AG	106 (26.8)	73 (25.1)	
GG	48 (12.1)	32 (11.0)	
*GPIIIa* (rs11871251)			
AA	151(38.1)	96 (33.0)	0.473
AG	135 (34.1)	105 (36.1)	
GG	110 (27.8)	90 (30.9)	
Platelet aggregation (%)			
AA-induced	87.9 ± 15.6	82.2 ± 13.8	<0.001
ADP-induced	88.1 ± 17.2	83.5 ± 12.9	<0.001
Platelet-leukocyte aggregates (%)			
Leukocyte	24.3 ± 7.3	21.6 ± 7.1	<0.001
Neutrophil	24.2 ± 7.2	22.1 ± 6.2	<0.001
Monocyte	24.9 ± 6.8	21.8 ± 5.3	<0.001
Lymphocyte	23.8 ± 7.2	22.3 ± 6.2	0.007

### Clinical characteristics in patients with and without carotid plaque

Among 396 patients (276 were atherothrombotic [AT] stroke, and 120 were small artery disease [SAD] stroke), 294 (74.2%) patients had plaque (188 had stable plaque [SP], 106 had vulnerable plaque [VP]). Diabetes mellitus, hypertension, AT stroke, and hyperlipidemia were more frequent in patients with plaque than those patients without plaque. Compared with patients with SP, the frequency of hypertension, diabetes mellitus and hyperlipidemia was higher in patients with VP (Table 2). The platelet–leukocyte aggregates and platelet aggregation were significantly higher in patients with VP compared with patients without plaque or with SP (Table 2).

**Table 2:** Clinical characteristics, platelet aggregation and platelet-leukocyte aggregates in patients with or without carotid plaque

Characteristics	VP (*n* = 106)	SP (*n* = 188)	Non- plaque (*n* = 102)	*P* value
Age (years)	68.6 ± 10.8	68.1 ± 11.9	67.9 ± 11.9	0.535
Men (*n*, %)	62 (58.5)	112 (59.6)	61 (59.8)	0.912
Hypertension (*n*, %)	95 (89.6)	140 (74.5)	52 (51.0)	<0.001
Diabetes mellitus (*n*, %)	50 (47.2)	64 (34.0)	24 (23.5)	0.008
Previous MI (*n*, %)	1 (0.9)	2 (1.1)	2 (2.0)	0.986
Current smoking (*n*, %)	45 (42.5)	75 (39.9)	40 (39.2)	0.898
Alcohol intake (*n*, %)	49 (46.2)	86 (45.7)	45 (44.1)	0.978
Body mass index (kg/m2)	24.1 ± 2.5	24.0 ± 2.6	23.9 ± 2.6	0.582
Hyperlipidemia (*n*, %)	82 (77.4)	124 (65.9)	56 (54.9)	0.004
Fasting blood glucose (mM)	7.1 ± 2.1	7.0 ± 2.1	6.9 ± 2.4	0.315
Hemoglobin A1c (%)	6.2 ± 1.4	6.1 ± 1.4	6.0 ± 1.5	0.136
Homocysteine (mM)	14.3 ± 4.3	13.9 ± 4.2	13.5 ± 4.1	0.138
Stroke subtype (*n*, %)				
AT stroke	78 (73.6)	135 (71.8)	63 (61.8)	0.046
SAD stroke	28 (26.4)	53 (28.2)	39 (38.2)	-
Previous or ongoing drug				
treatments (*n*, %)				
Antihypertensive drugs	32 (30.2)	57 (30.3)	32 (31.4)	0.893
Hypoglycemic drugs	29 (27.4)	47 (25.0)	19 (18.6)	0.127
Statins	14 (13.2)	25 (13.3)	12 (11.8)	0.336
Antiplatelet drugs	21 (19.8)	39 (20.8)	23 (22.6)	0.875
Platelet aggregation (%)				
AA-induced	91.2 ± 11.4	86.9 ± 10.4	84.6 ± 11.7	0.002
ADP-induced	89.7 ± 12.1	86.1 ± 11.5	84.7 ± 10.8	0.008
Platelet-leukocyte aggregates (%)				
Leukocyte	28.1 ± 6.7	23.8 ± 5.4	22.8 ± 7.2	<0.001
Neutrophil	27.2 ± 6.8	22.9 ± 7.1	22.2 ± 6.4	<0.001
Monocyte	26.9 ± 5.5	22.3 ± 6.3	21.8 ± 4.7	<0.001
Lymphocyte	26.4 ± 5.6	22.4 ± 7.5	21.8 ± 5.8	<0.001

VP, vulnerable plaque; SP, stable plaque; MI, myocardial infarction; AT, atherothrombotic; SAD, small artery disease; AA, arachidonic acid; ADP, adenosine diphosphate.

### Genotype distributions in patients with and without plaque

The frequency of *TXA2R* rs1131882TT, *TXAS1* rs2267679TT, *TXAS1* rs41708TT, *P2Y12* rs16863323T, and *GPIIIa* rs2317676GG was higher in patients with plaque than without plaque, or in patients with VP than with SP using a single-locus analytical approach (Table 3). However, the genotype of rs1131882TT, rs2267679TT, rs41708TT, rs16863323TT, and rs2317676GG was not independently associated with risk of VP after adjusting for potential confounding variables.

**Table 3:** Genotype distribution comparison among the three groups (*n,* %)

	VP (*n* = 106)	SP (*n* = 188)	Non- plaque (*n* = 102)	*P* value
*TXA2R* (rs1131882)				
CC	28 (26.4)	65 (34.6)	42 (41.2)	0.009
CT	47 (44.3)	87 (46.3)	50 (49.0)	
TT	31 (29.2)	36 (19.1)	10 (9.8)	
*TXAS1* (rs2267679)				
CC	2 (1.9)	8 (4. 3)	1(0. 9)	<0.001
CT	14 (13.2)	42 (22.3)	35 (34.3)	
TT	90 (84.9)	138 (73.4)	66 (64.7)	
*TXAS1* (rs194149)				
AA	17 (16.0)	33 (17.5)	15 (14.7)	0.916
AG	53 (50.0)	90 (47.9)	54 (52.9)	
GG	36 (34.0)	65 (34.6)	33 (32.4)	
*TXAS1* (rs41708)				
GG	53 (50.0)	114 (60.6)	72 (70.6)	<0.001
GT	30 (28.3)	55 (29.3)	25 (24.5)	
TT	23 (21.7)	19 (10.1)	5 (4.9)	
*P2Y1* (rs701265)				
AA	58 (54.7)	102 (54.3)	57 (55.9)	0.986
AG	32 (30.2)	57 (30.3)	30 (29.4)	
GG	16 (15.1)	29 (15.4)	15 (14.7)	
*P2Y1* (rs1439010)				
AA	56 (52.8)	103 (54.8)	56 (54.9)	0.967
AG	34 (32.1)	57 (30.3)	31 (30.4)	
GG	16 (15.1)	28 (14.9)	15 (14.7)	
*P2Y1* (rs1371097)				
CC	53 (50.0)	100 (53.2)	55 (53.9)	0.926
CT	35 (33.0)	59 (31.4)	31 (30.4)	
TT	18 (17.0)	29 (15.4)	16 (15.7)	
*P2Y12* (rs16863323)				
CC	26 (24.5)	55 (29.3)	40 (39.2)	0.022
CT	30 (28.3)	65 (34.5)	36 (35.3)	
TT	50 (47.2)	68 (36.2)	26 (25.5)	
*P2Y12* (rs9859538)				
GG	72 (67.9)	131 (69.7)	70 (68.6)	0.946
AG	23 (21.7)	40 (21.3)	24 (23.5)	
AA	11 (10.4)	17 (9.0)	8 (7.8)	
*GPIIIa* (rs2317676)				
AA	62 (58.5)	114 (60.6)	66 (64.7)	0.003
AG	21 (19.8)	54 (28.7)	31 (30.4)	
GG	23 (21.7)	20 (10.6)	5 (4.9)	
*GPIIIa* (rs11871251)				
AA	40 (37.7)	72 (38.3)	39 (38.2)	0.953
AG	35 (33.0)	64 (34.0)	36 (35.3)	
GG	31 (29.2)	52 (27.7)	27 (26.5)	

VP, vulnerable plaque; SP, stable plaque.

### Gene-gene interaction and its association with carotid plaque vulnerability

The associations of gene-gene interactions among the 11 variants with carotid plaque vulnerability were investigated using GMDR approach (Table 4). The best model for VP including rs1131882, rs2317676 and rs16863323 scored 10/10 for cross-validation consistency and 9/10 for sign test after adjustment with covariates (*P* = 0.015, Table 4). The *P* value for prediction error was 0.028 for GMDR using permutation testing.

**Table 4:** Comparison of the best models, prediction accuracies, cross-validation consistencies, and *P* values for vulnerable plaque identified by GMDR

Best model^*^	Training balanced accuracy	Testing balanced accuracy	Cross-validation consistency	Sign test (*P*)
1	0.397	0.612	6/10	8 (0.423)
1, 2	0.525	0.605	9/10	8 (0.342)
1, 2, 3	0.687	0.672	10/10	9 (0.015)
1, 2, 3, 4	0.585	0.612	7/10	7 (0.325)
1, 2, 3, 4, 5	0.575	0.512	8/10	6 (0.642)
1, 2, 3, 4, 5, 6	0.622	0.479	7/10	8 (0.576)
1, 2, 3, 4, 5, 6, 7	0.499	0.526	8/10	5 (0.734)
1, 2, 3, 4, 5, 6, 7, 8	0.613	0.611	7/10	6 (0.412)
1, 2, 3, 4, 5, 6, 7, 8, 9	0.598	0.572	6/10	7 (0.665)
1, 2, 3, 4, 5, 6, 7, 8, 9, 10	0.613	0.476	8/10	5 (0.684)
1, 2, 3, 4, 5, 6, 7, 8, 9, 10, 11	0.504	0.644	9/10	6 (0.337)

^*^rs1131882, rs2317676, rs16863323, rs194149, rs2267679, rs41708, rs701265, rs1439010, rs1371097, rs9859538, rs11871251 are symbolized as 1–11, respectively.

GMDR, generalized multifactor dimensionality reduction.

### Different genotype combinations and the risk of carotid plaque vulnerability

Subsequently, we assessed the associations of different genotype combinations among rs1131882, rs2317676, and rs16863323 with VP risk. Compared to patients harboring rs1131882CC, rs2317676AA, and rs16863323CC (wild-type genotypes), the relative risk of different genotype combinations of rs1131882, rs2317676 and rs16863323 was analyzed. The three genotype combinations making larger contributions to VP risk were rs1131882TT, rs2317676GG and rs16863323TT; rs1131882TT, rs2317676AG and rs16863323CT; and rs1131882TT, rs2317676GG and rs16863323CT/TT (Table 5), and were defined as the high-risk interactions. The other genotype combinations among rs1131882, rs2317676, and rs16863323 did not reach cut-off significance level of 0.05, and were defined as the low-risk interactions (Table 5).

**Table 5:** Associations between genotype combinations and vulnerable plaque

rs1131882	CC	TT	TT	TT	CT	TT	TT, CT	TT, CT
rs2317676	AA	GG	AG	GG	AG	GG, AG	GG	GG, AG
rs16863323	CC	TT	CT	CT, TT	CT	TT	TT	TT, CT
OR	1^*^	2.83	2.26	2.12	1.31	1.28	1.05	1.08
95% CI	–	1.35–8.32	1.16–6.48	1.13–5.25	0.94–2.56	0.83–2.02	0.67–1.78	0.79–1.83
*P* value	–	0.002	0.022	0.033	0.196	0.433	0.627	0.538

^*^The low-risk genotype for each genetic factor was used as the reference OR. OR, odds ratio; CI, confidence intervals.

### Logistic regression analysis of risk of carotid plaque vulnerability

The relative risk for VP conferred by different genotype combinations among rs1131882, rs2317676 and rs16863323 was assessed using multivariate logistic regression analysis. The high-risk interactions were assigned as one, and the low-risk interactions were assigned as zero. The other variables that showed a significant association with VP (*P* < 0.05) on univariate analysis, and previous statins or antiplatelet treatments were also entered in the multivariate logistic regression model. The results revealed that the high-risk interactions were independently associated with risk for VP after adjustment with covariates (OR, 2.61, 95% CI 1.33–7.98, *P* = 0.005, Table 6).

**Table 6:** Multivariate analysis of the major risk factors for vulnerable plaque

Risk factor	OR^*^	95% CI	*P* value
Hypertension	1.86	1.07–4.09	0.033
Diabetes mellitus	0.95	0.82–1.87	0.288
AT stroke	0.83	0.76–1.52	0.673
Hyperlipidemia	0.81	0.66–1.38	0.712
Platelet aggregation	0.72	0.67–1.17	0.735
Platelet-leukocyte aggregates	0.83	0.79–1.26	0.668
Statins	0.42	0.51–1.08	0.103
Antiplatelet drugs	0.51	0.58–1.12	0.134
*TXA2R* rs1131882TT	1.37	0.96–3.08	0.092
*TXAS1* rs2267679TT	1.22	0.91–2.15	0.178
*TXAS1* rs41708TT	1.33	0.93–2.46	0.139
*P2Y12* rs16863323TT	1.37	0.95–2.24	0.113
*GPIIIa* rs2317676GG	1.49	0.97–3.87	0.081
High-risk interactions	2.61	1.33–7.98	0.005

OR, odds ratios; CI, confidence interval; AT, atherothrombotic.

^*^OR for Platelet aggregation and Platelet-leukocyte aggregates means per 1- Standard Deviation increase.

### Effect of the high-risk interactive genotypes on platelet activation

There were no significant differences in AA or ADP - induced platelet aggregation and platelet-leukocyte aggregates among genotypes of the 11 variants on admission. However, the platelet-leukocyte aggregates and platelet aggregation were significantly higher in patients carrying the high-risk interactive genotypes than those patients without carrying the high-risk interactive genotypes (Table 7).

**Table 7:** Effect of high-risk interactive genotypes on platelet aggregation and platelet-leukocyte aggregates

	Platelet aggregation(%)		Platelet-leukocyte aggregates (%)			
	AA-induced	ADP-induced	Leukocyte	Neutrophil	Monocyte	Lymphocyte
High-risk interactive						
genotypes						
Yes (*n* = 92)	90.1 ± 10.7	88.6 ± 11.2	25.8 ± 5.2	26.6 ± 5.6	27.1 ± 4.9	26.3 ± 5.8
No (*n* = 304)	83.7 ± 15.2	84.5 ± 14.8	23.1 ± 7.2	23.4 ± 5.8	23.6 ± 7.5	24.5 ± 6.3
*P* value	<0.001	0.008	<0.001	<0.001	<0.001	0.009

AA, arachidonic acid; ADP, adenosine diphosphate.

## DISCUSSION

Platelet activation was associated the pathophysiology of atherogenesis and IS [15, 16, 18, 19]. Our previous studies have administrated that the variants of platelet activation-relevant genes (*TXA2R, GPIIIa, P2Y12, P2Y1, TXAS1*) not only increase the risk for IS, but also are associated with response to antiplatelet drugs and clinical adverse outcomes after IS [13, 22, 25, 27, 28]. However, the associations of the variants in platelet activation-relevant genes with carotid plaque vulnerability were not fully understood. Our current results showed the frequency of *TXA2R* rs1131882TT, *TXAS1* rs2267679TT, *TXAS1* rs41708TT, *P2Y12* rs16863323TT, and *GPIIIa* rs2317676GG were higher in patients with VP than patients with SP or without plaque using a single-locus analytical approach. After adjusting for potential confounding variables, these genotypes were not independently associated with risk of VP. However, we observed significant gene-gene interactions among variants of rs1131882, rs2317676 and rs16863323 using the GMDR approach. The high-risk interactions of the three variants were associated with platelet activation, and independently associated with the risk for VP.

These indicated that single-locus analytical approach (such as linkage analysis) seems unsuitable for genetic etiology of carotid plaque vulnerability. Atherosclerosis is a common complex disease, and does not follow Mendelian pattern of inheritance [29]. It is possible that genes contribute to the complex diseases only by interactions with other genes, and the effects of individual variant may be too small to be detected [30]. Our previous studies have administrated that the GMDR analysis may be helpful to understand complex genetic etiology of IS [22, 25, 27, 28]. However, few studies used the GMDR approach to investigate complex genetic risk for carotid plaque vulnerability.

The most noteworthy finding in current study was that there were interesting synergistic effects of gene-gene interactions on the risk of carotid plaque vulnerability and platelet activation via the GMDR methods. Variants among rs1131882, rs2317676, and rs16863323 were identified to interact together to influence the risk for carotid plaque vulnerability. The risk of carotid plaque vulnerability was increased by 2.61-fold in patients carrying the high-risk interactive genotypes than those without carrying the high-risk interactive genotypes.

Despite the pathophysiological significance of the interactions of the three variants is unclear, these findings are very interesting. Atherosclerosis is associated with chronic inflammatory. Platelet activation may play a key role in the pathophysiology of atherogenesis [18, 19]. The present results showed that the platelet-leukocyte aggregates and platelet aggregation were higher in patients with VP than patients without plaque or with SP, or in patients carrying high-risk interactive genotypes than those without carrying the high-risk interactive genotypes. Thus, one possible explanation for the three variants interactions is that they together participate in platelet activation. TXA2R and TXAS are important components in TXA2 function, and binding of TXA2 to TXA2R is crucial for platelet activation [31]. Some studies have shown *TXA2R* polymorphisms are associated with cerebral infarction and platelet activation [31, 32]. Glycoprotein IIIa and platelet membranes receptors play an important role in platelet activation and arterial thrombosis. The *GPIIIa* rs2317676 and *P2Y12* rs16863323 encode glycoprotein IIIa and platelet membranes receptors, respectively. Fontana *et al.* [33] has shown that a haplotype of the *P2Y12* receptor gene is associated with platelet aggregation. The *GPIIIa* rs2317676 had an effect on platelet aggregation in acute IS patients [22]. Thus, we reason that interactions among rs1131882, rs2317676 and rs16863323 could provide these individuals with higher platelet activation, thereby increasing the risk for carotid plaque vulnerability.

There has several potential limitations in this study. First, due to the relative small sample size and one-center study. Our findings must be validated in larger and multi-center studies. Second, although we genotyped multiple known functional variants in platelet activation-relevant genes, some rare functional variants were not investigated in this population. Thus, future studies involving a larger set of genetic variants should be detected to elucidate the effects of the full extent of gene-gene interactions on carotid plaque vulnerability pathogenesis. Third, carotid plaque vulnerability was assessed by ultrasound in this study. Although ultrasound can identify carotid plaques and determine the extent of stenosis, HR-MRI may provide more information regarding plaque composition and morphology [8]. Thus, it is necessary to assess carotid plaque vulnerability using HR-MRI, and validate our finding in future. Finally, we aim of present study was to investigate the association between variants in platelet activation-relevant genes and risk for carotid plaque vulnerability. Thus, we did not investigate the relations between these variants and carotid artery stenosis in this study.

## MATERIALS AND METHODS

### Study population

We consecutively enrolled 396 IS patients who had their first strokes and were admitted tothe People’s Hospital of Deyang City within 72 h of the onset of stroke between August 2010 and March 2013. IS in all cases was due to AT and SAD according to the Trial of ORG 10172 in the Acute Stroke Treatment classification system [34]. The detailed procedures for the recruitment of IS patients, inclusion criteria and exclusion criteriaweredescribed in our previous article [14]. The healthy volunteers who served as controls were selected from outpatients without history of stroke and carotid plaque as confirmed by medical history as well as physical and laboratory examinations at our center. This study protocol was reviewed and approved by the Ethics Committee of our hospital. Each of the participants provided written informed consent before enrollment into this study. Vascular risk factors, including age, gender, current smoking, alcohol intake, body mass index, history of diabetes mellitus, hypertension and myocardial infarction [MI] were recorded. Fasting blood samples were tested for blood sugar, hemoglobin A1c, triglycerides (TG), total plasma cholesterol (TC), low-density lipoprotein cholesterol (LDL-C), and homocysteine. Hyperlipidemia was defined as TC > 200 mg/dL, TG > 180 mg/dL or use of lipid-lowering medication.

### Carotid plaque vulnerability assessment by B-mode ultrasound

Bilateral common and internal carotid arteries, as well as bifurcations, were examined for atherosclerotic plaque presenceusing a diagnostic ultrasound device (type 512, Acuson Sequoia Apparatus, 7.5-MHz probe, Berlin, Germany) in all patients, according to standard scanning and reading protocols [7]. Assessment of plaque morphology was performed with the use of criteria established at an international consensus meeting on the morphology and risk of carotid plaques [35]. Plaque echogenicity was graded as uniformly anechoic, isoechoic, or hyperechoic; predominantly anechoic, isoechoic, or hyperechoic; or unclassifiable calcific. Plaque surface structure was assessed as smooth, irregular, or ulcerated. According to plaque echogenicity and surface structure, carotid plaque was further classified into I to IV [14, 35]. Plaque of class I or class II was defined as VP, and plaque of class III or class IV was defined as SP. Plaquemorphology and surface structure were graded independently by both authors blinded to patient clinical status. For a test run in this study, we assessed the reproducibility of plaque echogenicity in 33 randomly selected plaques. Intraobserver and interobserver coefficients of variation for plaque echogenicity were 8.2% and 8.8%, respectively, suggesting relatively reliable measurements in current study. The detailed procedures for evaluating plaques, types of plaques, intraobserver and interobserver coefficients were performed as described in our previous study [14]. According to the results of carotid ultrasonography, the patients were divided into three groups: non-carotid plaque group, VP group and SP group. Furthermore, 40 patients with ultrasound-based carotid plaque were also measured blindly by HR-MRI. Exact agreement of VP was found in 92% of the cases, and there were no major disagreements. These indicated that carotid ultrasound is a relatively reliable method for assessing carotid plaque vulnerability.

### Genotyping

The 11 SNPs of platelet activation-relevant genes were selected from the NCBI database (http://www.ncbi.nlm.nih.gov/SNP), according to the criteria: (1) the SNPs had been assessed in previous studies; (2) the SNPs lead to amino acid changes; (3) the SNPs with minor allele frequency >0.05; (4) Tagging SNPs across different human populations (http://pga.gs.washington.edu).

Whole blood (3 mL) was drawn from an arm vein into a sterile tube containing ethylenediaminetetraacetic acid (EDTA) and stored at –80° C until genotype analysis was performed.Genotypes of the 11 variants were examined using matrix-assisted laser desorption ionization time-of-flight mass spectrometry methods as our previously described [13, 14, 22].

### Measurement of platelet activation

Venous blood (6mL) was drawn from an antecubital vein in each patient on admission. Platelet-leukocyte aggregates were measured by FC 500 MPC flow cytometry (Beckman Coulter Ltd, Krefeld, Germany), and we used direct fluorescent markers (all commercially available; Coulter Immunotech, Krefeld, Germany). Platelet aggregation was measured by light transmittance aggregometry. The results of optical platelet aggregometry are presented as the amplitude of light transmittance at five minutes after addition of the agonist 0.5 mM AA and 10 μM ADP with a BioData PAPS-4 platelet aggregometer (Helena Laboratories, Beaumont, TX, USA). The procedures for measuring platelet-leukocyte aggregates and platelet aggregation were described in our previous studies [36, 37].

### Statistical analysis

We calculated the sample size, based on a suggested sample size requirement of gene-gene interactions [38]. We calculated that a sample of 180 patients with SP and 100 patients with VP would sufficiently provide 80% power at the 5% significance level calculated using three genetic models: the dominant model, the additive model, and the recessive model.

SPSS 16.0 (SPSS Inc., Chicago, IL, USA) was used to perform the statistical analyses. The χ^2^ test was used to analyze the deviation of Hardy–Weinberg equilibrium for genotype frequencies. Continuous variables were compared using analysis of variance (ANOVA) followed by Student-Newman-Keuls test, and discrete variables were assessed using the χ^2^ testamong patients with VP, SP and non-plaque. Difference of genotype frequencies among patients with VP, SP and non-plaque was also compared by χ^2^-test. Gene-gene interaction was assessed using the GMDR method, as described in our previous studies [21, 24]. The GMDR v 0.7 program was used in this study (www.healthsystem. virginia.edu/internet/addiction-genomics/Software) [26]. This model was then confirmed by permutation test implemented in the GMDR software. Subsequently, logistic regression analysis was performed to adjust covariate risk factors using variables with *P* values < 0.05 in univariate analysis, and previous statins or antiplatelet treatments to assess the independent contribution of the variants and interactions among these variants on carotid plaque vulnerability, and odds ratio (OR) with 95% confidence intervals (CI) were calculated. All tests were two sided, and the threshold level of *P* < 0.05 denoted statistical significance.

## CONCLUSIONS

In present study, the 11 variants in platelet activation-relevant genes were not associated with risk of carotid plaque vulnerability after adjusting for potential confounding variables. However, the GMDR analysis showed that there were synergistic effects of gene-gene interactions among *TXA2R*r s1131882, *GPIIIa* rs2317676 and *P2Y12* rs16863323 on carotid plaque vulnerability risk. However, our current findings are needed to be validated in future studies.

## Abbreviations

IS: ischemic stroke
TXA2: thromboxanesA2
TXAS: thromboxane synthase
GPIIb: glycoprotein IIb
GPIIIa: glycoprotein IIIa
TXA2R: thromboxanesA2receptor
P2Y12: platelet membranes receptors
SNPs: single nucleotide polymorphisms
GMDR: generalized multifactor dimensionality reduction
AT: atherothrombotic
SAD: small artery disease
MI: myocardial infarction
TC: total cholesterol
TG: triglycerides
LDL-C: low-density lipoprotein cholesterol
VP: vulnerable plaque
SP: stable plaque
AA: arachidonic acid
ADP: adenosine diphosphate
OR: odds ratio
CI: confidence interval
